# Does a spinal implant alter dual energy X-ray absorptiometry body composition measurements?

**DOI:** 10.1371/journal.pone.0222758

**Published:** 2019-09-19

**Authors:** Pei-Lin Hsiao, Shu-Feng Hsu, Po-Han Chen, Hsiao-Wei Tsai, Hsin-Ying Lu, Yue-Sheng Wang, Li-Wen Lee

**Affiliations:** 1 Department of Diagnostic Radiology, Chang Gung Memorial Hospital, Chiayi, Taiwan; 2 Department of Nursing, Chang Gung Memorial Hospital, Chiayi, Taiwan; 3 Department of Orthopedic Surgery, Chang Gung Memorial Hospital, Yunlin, Taiwan; 4 Department of Nursing, Chang Gung University of Science and Technology, Chiayi Campus, Taiwan; United States Military Academy, UNITED STATES

## Abstract

**Background:**

Most manufacturer manuals do not verify the use of dual energy X-ray absorptiometry for body composition analysis in subjects with a metal implant. This study aimed to quantify the effects of a spinal implant on body composition, and to determine whether unadjusted lean mass estimates are valid for patients with a spinal implant.

**Methods:**

A total of 30 healthy subjects were recruited. Three consecutive scans were performed for each participant, one with and two without extraneous spinal implant, without repositioning between scans. Lean, fat and bone estimates in the total body, trunk and limb were measured.

**Results:**

Precision errors for all total and regional body compositions were within the recommended ranges. Bone masses in the trunk and total body were significantly increased with spinal implant, and the increases exceeded the least significant change. For total and regional lean and fat estimates, the measurements between subjects with and without metal implants were in substantial to almost perfect agreement and the differences were not significant and did not exceed the least significant change.

**Conclusions:**

Spinal metal artifacts significantly increased the total body and trunk bone mass but the differences in lean- and fat-related estimates at total and regional body levels and all estimates in the extremity remained within the clinical acceptable range. Thus, a spinal implant may not compromise screening of patients for fat and lean masses using dual energy X-ray absorptiometry. Application of image reconstruction or a filtering algorithm may help reduce the effect of metallic artifacts and further study is needed.

## Introduction

Dual energy X-ray absorptiometry (DXA), with its reasonable accuracy, accessibility and cost, has become a common tool for assessing body composition in both clinical and research settings. The determination of body composition using DXA exploits the principle that X-rays at two difference energies can decompose a sample into two tissue components with known mass attenuation coefficients [1]. DXA can also act as a three-component body composition method for assessing lean, fat and bone masses using specific soft tissue algorithm [2–4]. Owing to the DXA methodology, high density objects such as foreign bodies or metal prostheses in the scan areas may affect the partition ability on DXA images and are regarded as a relative contraindication for DXA analysis.

The influence of metal artifacts on DXA bone densitometry has been extensively investigated, and the most common solution for high density artifacts is to exclude the affected regions from the analysis. However, there is no universal consensus on which method to reduce the metal artifacts for DXA body composition analysis. In the literature, half-body scan has been suggested for obese subjects whose body dimensions exceed the width of the scanning area [5]. This method assumes bilateral symmetry of the body such that body composition will be interchangeable for either side. This method has also been used to replace the body composition data from the unilateral extremity with high density artifacts or implants with data from the contralateral extremity [6]. Although this method works well for artifacts in peripheral body parts, it does not work for those within the midline of the body, i.e., spinal pedicle screws or vertebral cement augmentation.

Axial skeletal prostheses such as spinal implants are located within the central region on a DXA image and theoretically, they may affect body composition analysis in the trunk region but may affect the results in the extremity to a lesser extent. However, there is a lack of validation studies for the application of DXA analysis in the presence of spinal implants. To the best of our knowledge, there is only one previous study quantified the effects of spinal implants on body composition by DXA [7]. In their study, the lean and fat masses showed statistically significant increases after an extraneous spinal implant (approximately 100 g), but the increases did not exceed least significant change (LSC). Since their study investigated only body composition estimate at the total body level in a small group of subjects (n = 7), further investigated is still needed. Therefore, this study aimed to investigate the effects of an extraneous spinal implant on the total and regional body composition measurements statistically and clinically, and to determine whether unadjusted body composition estimates are valid for patients with spinal implant.

## Methods

### Subjects

Subjects were recruited via hospital advertisements and word of mouth. Healthy adults of both sexes were recruited. Exclusion criteria were subjects with anatomical defects, un-removable scan artifacts in their bodies except for dental metal, body size exceeding the scanner field and females with actual or suspected pregnancy. The detailed recruitment criteria are shown in **S1 Table**.

### Study protocol

**Fig 1:**
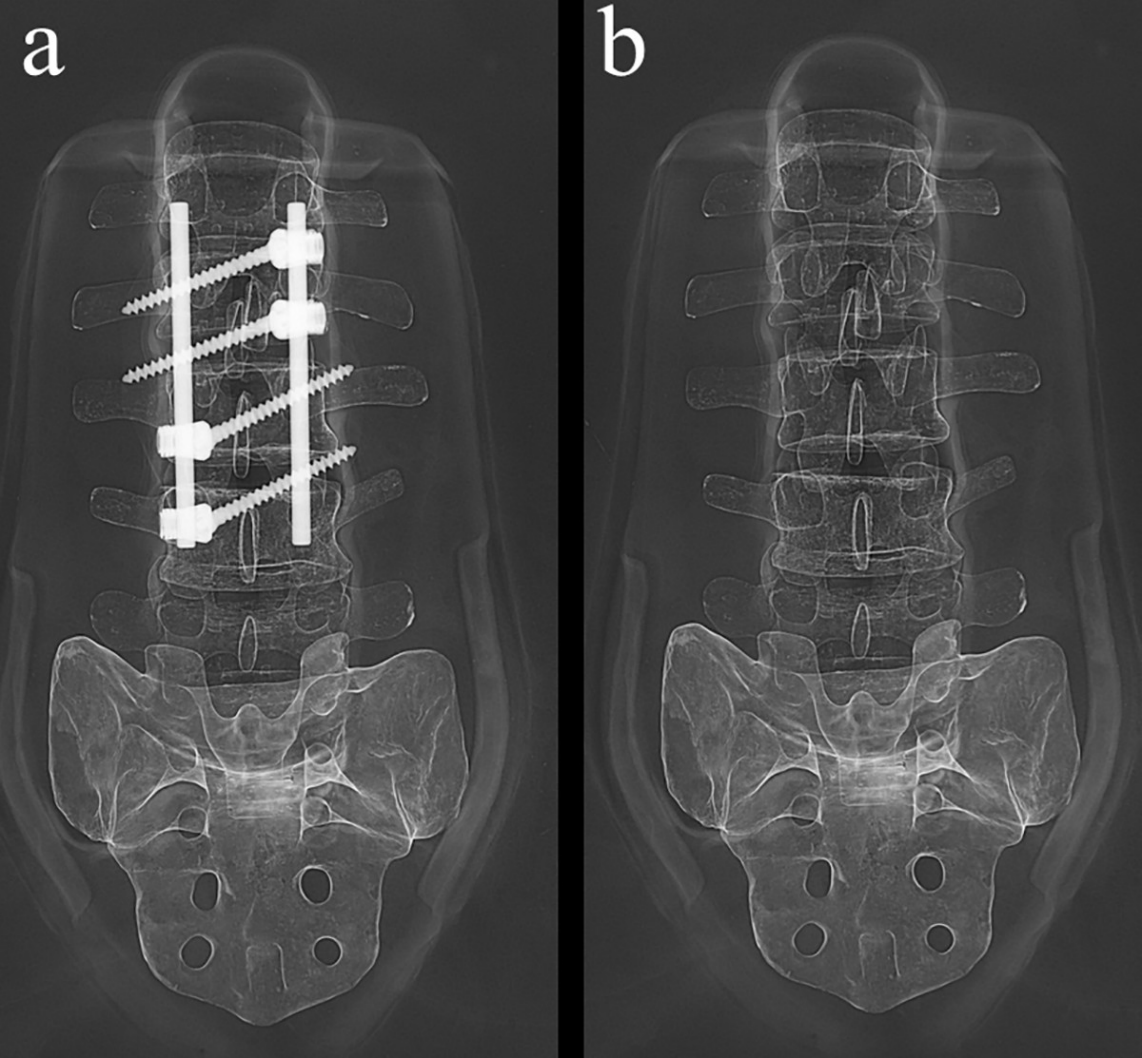
Illustration of spinal implants inserted on the back of an X-ray phantom.

This prospective cross-sectional study was approved by the Institutional Review Board of the Chang Gung Medical Foundation (No: 201800152A3) and conducted from April 2018 to January 2019. All participants gave their written informed consent. There was no dietary restriction prior to the study. On arrival, participants were asked to void and change into a light hospital gown. Body weight and height were measured using a digital scale. Three consecutive DXA scans were performed for each participant by the same technician. Subjects were instructed not to move between scans to avoid errors arising from repositioning. The first DXA scan was performed with a titanium spinal fixation placed under the back of subjects at the L1-4 levels (**Fig 1**). The second and third scans were acquired after careful removal of the spinal fixation, avoiding participant motion and repositioning between scans. The titanium spinal implant consisted of 2 rods and 4 screws with a total weight of 86.2 g. The total study time was about half an hour.

### Dual-energy X-ray absorptiometry (DXA)

Body composition was assessed using a fan-beam DXA system (Horizon W, Hologic, Inc.) equipped with Hologic Apex version 5.6, with the National Health and Nutrition Examination Survey (NHANES) body composition correction enabled. The scanner produces two different energy levels at 100 and 140 kVp. Periodic air scan without any scan object in the field was performed according to manufacturer guidelines for system maintenance. The whole body DXA scan took approximately 6 min with an effective radiation dose of 8.4 uSv [8]. All scanning and analysis were performed by the same radiology technologist with five years of experience in DXA body composition analysis. Placement of the sub-region cut lines on the two-dimensional DXA images was done in accordance with the manufacturer’s instructions [9]. Whole body and regional composition estimates, including lean, fat, and bone mineral content (BMC), were analyzed. Appendicular lean mass (ALM) was calculated as the sum of lean masses in the four extremities. Appendicular lean mass index (ALMI) was calculated by dividing ALM by height squared in meters.

### Statistical analysis

The first and second DXA scans were used to calculate body composition estimates in the presence and absence of a spinal implant. The second and third DXA scans were used to calculate the precision errors of DXA scans without repositioning the subjects. LSC was used to report the difference in two measures with the 95% confidence range. According to the recommendation by the International Society for Clinical Densitometry (ISCD), a sample size of 30 subjects was used for this study [10, 11]. The pairs of data for the 30 subjects were used to calculate the root mean square standard deviation (RMS SD) or coefficient of variation (CV) and LSC using a precision calculating tool provided by the ISCD (https://www.iscd.org/resources/calculators/). LSC was calculated as: LSC = 2.77 × CV. Variation of change was considered significant when the difference between measurements was equal to or greater than the LSC. Lin’s concordance correlation (CCC) was used to assess agreement between measurements using web-based calculator (www.niwa.co.nz/node/104318/concordance) [12]. The CCC coefficient (Rc) was interpreted as almost perfect (R_c_ > 0.99), substantial (0.99 ≥ R_c_ > 0.95), moderate (0.95 ≥ R_c_ > 0.9) and poor (R_c_ ≤ 0.9). All other analyses were carried out using IBM SPSS Statistics version 22.0. Paired t-test was used to calculate the difference between two measurements for the same subject. Pearson’s correlation coefficient (R) was used to examine the strength of linear relationship between two measures. Intraclass correlation (ICC) with two-way mixed model was used to evaluate the absolute agreement between measurements with and without a spinal implant [13]. The ICC coefficient (r) was interpreted as excellent (r > 0.9), good (0.9 ≥ r > 0.75), moderate (0.75 ≥ r > 0.5) and poor (r ≤ 0.5). Bland-Altman plot was used to calculate percentage difference between a pair of measurements against the mean of the pair [14]. Two-tailed p values < 0.05 were taken as statistically significant. Data were presented as mean ± standard deviation (SD).

## Results

Thirty healthy subjects (15 men and 15 women) were recruited into the study. Subject characteristics are shown in **Table 1**. Total body mass measured by DXA was very strongly correlated to body weight measured by digital scale in the absence of spinal implant (R = 0.997) with a very small standard error of the estimate of 0.768 kg.

**Table 1:** Subject characteristics.

	Female (n = 15)	Male (n = 15)
Age (yr)	37.5±9.4	32.3±10.5
	(26–58)	(21–61)
Height (cm)	161.0±6.9	174.2±4.3
	(149.7–170.4)	(170–182.9)
Weight (kg)	58.6±9.8	78.0±13.1
	(38.8–74.0)	(65.2–107.0)
BMI (kg/m^2^)	22.5±3.1	25.7±4.5
	(17.3–27.7)	(20.6–35.3)

Note: Data were presented as mean ± standard deviation (range). **Abbreviation:** BMI, body mass index.

Precision assessments of body composition estimates without subject repositioning between DXA scans are shown in **Table 2**. DXA precision was different for each body composition estimate. Precision errors were 1.09%, 0.97%, 1.73%, 1.73% and 0.64% for total BMC, lean, fat, percentage body fat (PBF) and bone mineral density (BMD), respectively. For skeletal muscle estimates, precision errors were 1.08% and 1.14% for ALM and ALMI, respectively. Fat-related estimates including fat mass and PBF had the highest precision errors, ranging 1.73–2.08%.

**Table 2:** Precision assessments in 30 subjects without spinal implant and without reposition between scans.

	Measurement 1		Measurement 2		Precision		LSC (95%CI)	
	Mean±SD	Range	Mean±SD	Range	RMS SD	CV (%)	RMS SD	CV (%)
**Total body**								
BMC (g)	2470±458	1542–3319	2463±446	1498–3273	26.855	1.09	74.389	3.02
Lean (g)	42047±10138	23617–61923	41948±10125	22911–61585	405.103	0.97	1122.136	2.67
Fat (g)	22325±6715	13019–40261	22374±6773	13646–41641	386.329	1.73	1070.130	4.79
PBF (%)	33.4±6.5	20.3–48.5	33.5±6.6	20.9–48.3	0.579	1.73	1.603	4.79
BMD (g/cm^2^)	1.216±0.099	1.023–1.402	1.213±0.095	1.005–1.400	0.008	0.64	0.021	1.76
**Extremities**								
BMC (g)	1199±291	691–1714	1195±283	684–1699	17.031	1.42	47.175	3.94
ALM (g)	18418±5404	9585–28646	18374±5441	9280–28633	198.917	1.08	551.000	3.00
Fat (g)	10118±2746	5605–16635	10106±2724	5709–16927	194.875	1.93	539.804	5.34
ALMI (kg/m^2^)	6.4±1.4	4.3–9.7	6.4±1.4	4.1–9.7	0.073	1.14	0.203	3.16
**Trunk**								
BMC (g)	644±129	354–865	646±125	357–851	7.491	1.16	20.750	3.22
Lean (g)	20531±4469	11666–29535	20467±4403	11223–29196	264.012	1.29	731.312	3.57
Fat (g)	11009±4093	4995–21961	11059±4180	5250–23034	229.130	2.08	634.690	5.75
PBF (%)	33.7±7.2	20.0–50.3	33.8±7.2	21.2–49.9	0.684	2.03	1.896	5.63

**Abbreviations:** ALM, appendicular lean mass; ALMI, appendicular lean mass index; BMC, bone mineral content; BMD, bone mineral density; CI, confidence interval; CV, correlation of variation; LSC, least significant change; PBF, percentage body fat; RMS SD, root mean square standard deviation; SD, standard deviation.

A subset of subjects was recruited to calculate precision error and LSC with repositioning of subjects between scans by the same technician (**S2 Table**). In that study, 15 healthy subjects were recruited and three consecutive DXA scans were performed with repositioning between scans. With subject repositioning, precision errors were 0.98%, 1.31%, 2.05%, 2.05% and 0.96% for total BMC, lean, fat, PBF and BMD, respectively (**S3 Table**). For skeletal muscle estimates, precision errors were 1.61% and 1.60% for ALM and ALMI, respectively (**S3 Table**). In general, precision errors for fat mass and PBF estimates were greater than those for lean and bone estimates at both the total and regional levels. Additionally, the estimated precision errors were greater for all total body and regional body composition estimates with subject repositioning compared to those without repositioning, except for total body BMC (**Table 2 and S3 Table**).

Body composition estimates with and without an extraneous spinal implant (86.2 g) are shown in **Table 3.** A variety of agreement tests were performed between these estimated, including paired t-test, ICC, CCC and Bland-Altman plot. For all lean- and fat-related estimates, the differences between subjects with and without metal implants were not significant (p > 0.05, **Table 3**) and did not exceed the LSC (**Tables 2 and 4**), indicating a non-significant effect of the spinal implant on these estimates (**Table 4**). Furthermore, these estimates were in almost perfect agreement, except for a substantial agreement by CCC (Rc = 0.989) in trunk PBF, and the limits of agreements were small and not of clinical importance (**Table 3**). Similar findings were noted for all body composition estimates with and without an extraneous spinal implant in the extremity which showed almost perfect agreement (r ≥ 0.998 by ICC and Rc ≥ 0.995 by CCC, **Table 3**) with small and not of clinical importance bias (-0.794% to 0.361%, **Table 3**). The differences were not statistically significant (p > 0.05, **Table 3**) and did not exceed LSC (**Tables 2 and 4**).

**Table 3:** Agreement between body composition estimates with and without spinal implant.

	Without implant		With implant					Bland-Altman Plot	
	Mean±SD	Range	Mean±SD	Range	p	ICC	CCC	Bias (%)	LOA (%)
**Total body**									
BMC (g)	2470±458	1542–3319	2665±454	1681–3497	<0.001	0.955	0.911	7.825	4.074 to 11.580
Lean (g)	42047±10138	23617–61923	41983±10191	22989–61161	0.521	0.999	0.999	-0.221	-2.937 to 2.496
Fat (g)	22325±6715	13019–40261	22263±6641	12590–39849	0.549	0.998	0.996	-0.192	-5.638 to 5.254
PBF (%)	33.4±6.5	20.3–48.5	33.3±6.5	19.3–47.3	0.601	0.996	0.991	-0.300	-5.707 to 5.107
BMD (g/cm^2^)	1.216±0.099	1.023–1.402	1.305±0.094	1.102–1.475	<0.001	0.817	0.684	7.116	3.901 to 10.330
**Extremities**									
BMC (g)	1199±291	691–1714	1197±286	690–1735	0.653	0.999	0.998	-0.053	-2.830 to 2.724
ALM (g)	18418±5404	9585–28646	18376±5366	9425–27937	0.489	0.999	0.996	0.361	-3.063 to 3.784
Fat (g)	10118±2746	5605–16635	10031±2687	5281–16280	0.076	0.998	0.995	-0.794	-6.169 to 4.581
ALMI (kg/m^2^)	6.4±1.4	4.3–9.7	6.4±1.4	4.2–9.4	0.496	0.998	0.997	-0.234	-3.665 to 3.197
**Trunk**									
BMC (g)	644±129	354–865	842±131	514–1031	<0.001	0.627	0.448	27.46	17.01 to 37.91
Lean (g)	20531±4469	11666–29535	20495±4534	11204–29326	0.590	0.998	0.997	-0.292	-4.060 to 3.476
Fat (g)	11009±4093	4995–21961	11020±4099	5266–21919	0.865	0.998	0.997	0.166	-6.512 to 6.844
PBF (%)	33.7±7.2	20.0–50.3	33.5±7.2	19.3–48.8	0.605	0.994	0.989	-0.365	-7.008 to 6.278

**Abbreviations:** ALM, appendicular lean mass; ALMI, appendicular lean mass index; BMC, bone mineral content; BMD, bone mineral density; CV, correlation of variation; CCC, Lin’s concordance correlation; ICC, intraclass correlation; LOA, limits of agreement; LSC, least significant change; PBF, percentage body fat; RMS SD, root mean square standard deviation; SD, standard deviation.

**Table 4:** Absolute and percentage differences in body composition estimates with and without spinal metal implant.

	Absolute difference				Percentage difference (%)			
	Mean	SD	Lower 95%CI	Upper 95%CI	Mean	SD	Lower 95%CI	Upper 95%CI
**Total body**								
BMC	194.9	36.4	181.3	208.4	8.16	2.07	7.39	8.94
Lean	-64.0	539.3	-265.3	137.4	-0.21	1.38	-0.73	0.31
Fat	-62.1	560.5	-271.3	147.2	-0.16	2.77	-1.19	0.88
PBF	-0.1	0.9	-0.4	0.2	-0.26	2.74	-1.29	0.76
BMD	0.089	0.018	0.082	0.096	7.39	1.76	6.74	8.05
**Extremities**								
BMC	-1.5	18.6	-8.5	5.4	-0.04	1.42	-0.57	0.49
ALM	-42.5	332.0	-166.4	81.5	-0.22	1.75	-0.87	0.43
Fat	-87.1	259.7	-184.1	9.8	-0.76	2.71	-1.77	0.26
SMI	0.0	0.1	-0.1	0.0	-0.22	1.75	-0.87	0.43
**Trunk**								
BMC	198.2	21.0	190.4	206.0	32.05	7.21	29.35	34.74
Lean	-36.0	362.4	-171.3	99.3	-0.27	1.92	-0.99	0.44
Fat	10.6	337.4	-115.4	136.6	0.22	3.41	-1.05	1.50
PBF	-0.1	1.1	-0.5	0.3	-0.31	3.37	-1.57	0.95

With an extraneous spinal implant, the total body BMC increased from 2470 g to 2665 g (p < 0.001, **Table 3**), the trunk BMC increased from 644 g to 842 g (p < 0.001, **Table 3**), whereas the limb BMC decreased from 1199 g to 1197 g (p = 0.653, **Table 3**). The mean difference in the total and trunk BMC estimates were 194.9 g (8.16%) and 198.2 g (32.05%), respectively (**Table 4**), which reached statistical significance and exceeded the LSC (**Table 2**). In contrast, the mean difference in the limb BMC was only -1.5 g (-0.04%), which did not reach statistical significance and did not exceed the LSC (**Table 2**). The trunk BMC estimated between subjects with and without a spinal implant were in moderate agreement by ICC (r = 0.627, **Table 3**) and poor agreement by CCC (Rc = 0.448, **Table 3**), and the limits of agreement were large (17.01% to 37.91%).

At the total body level, BMD estimates increased from 1.216 g/cm^2^ to 1.305 g/cm^2^ in the presence of a spinal implant, and the increase reached statistical significance (p < 0.001, **Table 3**) and exceeded the LSC (**Table 2**). Although in good agreement by ICC (r = 0.817, **Table 3**), the agreement between total body BMD with and without a spinal implant were in poor agreement by CCC (Rc = 0.684, **Table 3**) and the limits of agreement were large (3.901% to 10.33%). Thus, total BMD estimates were not acceptable to be used in the presence of a spinal implant.

## Discussion

Most DXA manufacturer manuals do not verify the use of DXA for body composition analysis in subjects with a metal implant. Generally, subjects with metal implants are excluded from recruitment in studies of body composition. As far as we know, no studies have yet addressed regional body composition changes in patients with spinal implants. In our study, both total body and regional body composition changes following the placement of an extraneous spinal implant were explored, showing that BMC estimates in the trunk and total body were significantly increased with an extraneous spinal implant but BMC estimates in the extremities were not affected. Moreover, the changes in the lean- and fat-related estimates at total and regional body levels were non-significant and remained within the clinical acceptable range. Our study suggested that axially located spinal implant may not influence the appendicular body composition estimates.

During DXA analysis, possible sources of error affecting the results may arise from the instrument, patient and operator [15, 16]. Instrument-related errors, such as equipment drift and precision errors, can be eliminated by regular quality assessment according to manufacturer’s guidelines [17]. Patient-related errors can be reduced by educating the patient about the DXA procedure to obtain the greatest cooperation from the patient. Since DXA scan requires the patient to lie still on the scanner table throughout the scan for a total of 3 to 20 min, all DXA images may have some degree of motion artifact such as respiration, cardiac motion, bowel peristalsis, and patient restlessness. Some movement may significantly interfere with DXA results and cause image degradation, depending on the degree and orientation of the movement [18]. Operator-related errors such as subject position and sub-regional line placement are one of the biggest sources of errors in DXA scan. In our study, instrument- and patient-related precision errors were calculated from repeat measurements without changing the subject position and sub-region lines. For comparison, precision errors according to the ISCD recommendation were also calculated from a subset of subjects repositioned between scans, to calculate the errors arising from instrument, patient and operator.

According to the 2015 ISCD Official Positions, the minimum acceptable precision for an individual technologist is 3%, 2% and 2% for total fat, lean mass, and PBF, respectively. In our study, precision errors were within the acceptable ranges with or without subject repositioning. As expected, the precision errors without subject repositioning between scans were smaller than those with subject repositioning for all body composition estimates, except for BMC in the total body and extremity. Operator-related errors were represented by the differences in precision errors in subjects with and without repositioning between scans. Unlike the general agreement, our study showed that the differences in precision errors with and without operator-related errors were small at the total body and regional measures Our scanner passed all the regular quality assurance tests and all DXA images were free of visibly appreciated motion artifact or image degradation. The reason for this discrepancy is not clear. Furthermore, the changes in lean and fat estimate with the presence of an extraneous spinal implant were small and insignificant compared to the calculated LSC with and without subject repositioning in our study. As a result, DXA may be extended to allow the study of skeletal muscle mass in patients with spinal implants.

Metal implants may appear as a greater bone mass in the projected image pixels in DXA analysis and cause an error in the data analysis. Giangregorio et al. reported a 61.4 g increase in whole body BMC with an extraneous 150.8 g stainless steel spinal rod [7]. Madsen et al. showed an increase of 150 g in leg BMC and 35 g in total BMC with a titanium hip prosthesis of 450 g [19]. Similar to previous studies, our study showed a significant increase in the total and trunk BMC when a titanium spinal implant was placed below the subject. However, the total weight of our spinal implant was 86.2 g, whereas the resulting increase in the total body BMC was up to 194.9 g. The reason for this greater increase in estimated BMC in our study remains unclear. It may due to the differences in the metallic materials, areas of the 2D projection and the post-processing algorithm used in these scanners.

An overestimation of lean mass with a metal implant has been previously reported [6, 7, 19]. Madsen et al. showed significant and pronounced increases in lean mass at both regional and whole body levels in 21 patients with a titanium hip prosthesis using a Norland scanner [19]. Giangregorio et al. showed a 0.7±0.5% increase in total lean mass in 7 patients with 100g of stainless steel spinal rods on top of the back using a Hologic 4500A scanner [7]. Although the increase was statistically significant, the changes did not exceed their institutional LSC. Di Monaco et al reported a significant increase in leg lean mass (difference between both legs 451 g) in the fractured leg of 313 women with hip prosthesis using a Hologic 4500W scanner [6]. In contrast to previous studies, our results showed that lean estimates were not affected by a titanium spinal implant. Possible explanations for the discrepancy include the use of different DXA models which involve different algorithms for soft tissue partition, the body regions which affected by the prosthesis, as well as the different experimental designs between studies.

Common DXA scanners include pencil-beam and fan-beam models. Fan-beam technology provides a short scan time but greater image magnification and distortion due to parallax errors [20, 21]. With fan-beam DXA, more than one fan-beam may pass through the metal implant, increasing the amount of artifact produced. It is possible that these artifacts may cause errors in processing projection data and image reconstruction. In this study, duplicate images of spinal implants were not produced and lean and fat masses in the trunk region and total body were not affected by the extraneous spinal implant. Our study suggests that spinal implants do not cause significant errors in image registration and data processing in the Hologic Horizon W scanner.

There are several limitations of the current study. Metallic artifacts may vary with the hardware composition, orientation and position within the scanner. Therefore, our results may only be applicable to subjects with titanium spinal implants and scanned by a Hologic fan-beam scanner. Second, the effect of a different amount of spinal implants on DXA analysis was not explored in this study. Third, this study investigated the effect of an extraneous but not an endogenous implant. Since DXA is a two-dimensional imaging modality, both extraneous and endogenous spinal implants may produce similar projected DXA image for further analysis. Forth, the sample size in our study was small. However, these results were obtained from assessments with 30 degrees of freedom which meet the minimal sample size requirements for precision study recommended by the ISCD.

## Conclusions

This study examined the effects of a spinal implant on body composition on a fan-beam DXA scanner. The results indicated that spinal metal artifacts significantly increased the total body and trunk BMC, whereas the lean, fat and percentage fat in the total body and body segments were small, insignificant and not of clinical importance. Our study suggested that DXA may be extended to allow the study of lean and fat estimates in patients with spinal implants.

## Supporting information

S1 Table Eligibility criteria for subject recruitment. (DOCX)

S2 Table Subjects characteristics. (DOCX)

S3 Table Precision assessment results. (DOCX)
