# Supplementary material for: Does a spinal implant alter dual energy X-ray absorptiometry body composition measurements?
Source: PLoS One. 2019 Sep 19;14(9):e0222758. doi: 10.1371/journal.pone.0222758 (PMC6752773; doi:10.1371/journal.pone.0222758)
Supplement: S1 Table — (DOCX) [file pone.0222758.s001.docx]

**Supplemental Table 1. Eligibility criteria for subject recruitment.**

| Inclusion criteria | Exclusion criteria |
| --- | --- |
| Healthy adults of both sexes | 1. Having body or limb defects due to congenital anomalies or injuries 2. Having medical devices or implants that interfere with scan accuracy 3. Wearing radiopaque objects that cannot be removed 4. Having had X-ray procedures using iodinated contrast within 3 days or barium within 2 weeks 5. Females with actual or suspected pregnancy 6. Body width ≥ 66 cm or body height ≥ 195.6 cm 7. Body weight ≥ 204 kg |
