# Supplementary material for: Does a spinal implant alter dual energy X-ray absorptiometry body composition measurements?
Source: PLoS One. 2019 Sep 19;14(9):e0222758. doi: 10.1371/journal.pone.0222758 (PMC6752773; doi:10.1371/journal.pone.0222758)
Supplement: S2 Table — (DOCX) [file pone.0222758.s002.docx]

**Supplementary Table 2. Subjects characteristics.**

| **Sex**  **(M:F)** | **Age (yr)**  **(range)** | **Height (cm)**  **(range)** | **Weight (kg)**  **(range)** | **BMI (kg/m^2^)**  **(range)** |
| --- | --- | --- | --- | --- |
| 4:11 | 38.5±12.4 | 162.0±7.2 | 62.3±9.3 | 23.2±2.4 |
|  | (25-62) | (153.5-180.0) | (50.6-84.7) | (20.1-28.4) |

Note: Mean ± standard deviation

**Abbreviation:** BMI, body mass index.
