# Supplementary material for: Does a spinal implant alter dual energy X-ray absorptiometry body composition measurements?
Source: PLoS One. 2019 Sep 19;14(9):e0222758. doi: 10.1371/journal.pone.0222758 (PMC6752773; doi:10.1371/journal.pone.0222758)
Supplement: S3 Table — (DOCX) [file pone.0222758.s003.docx]

**Supplementary Table 3. Precision assessment results.**

| **Results** | **Mean (±SD)** | **RMS SD** | **(%CV)** | **LSC** | **LSC (%)** |
| --- | --- | --- | --- | --- | --- |
| **Total body** |  |  |  |  |  |
| BMC (g) | 2115±341 | 20.647 | 0.98 | 57.191 | 2.70 |
| Lean (g) | 34787±6537 | 455.695 | 1.31 | 1262.275 | 3.63 |
| Fat (g) | 23073±3831 | 473.641 | 2.05 | 1311.985 | 5.69 |
| PBF (%) | 38.6±4.5 | 0.791 | 2.05 | 2.192 | 5.68 |
| BMD (g/cm^2^) | 1.147±0.087 | 0.011 | 0.96 | 0.030 | 2.65 |
| **Extremities** |  |  |  |  |  |
| BMC (g) | 980±205 | 12.942 | 1.32 | 35.848 | 3.66 |
| ALM (g) | 14521±3641 | 233.121 | 1.61 | 645.744 | 4.45 |
| Fat (g) | 10498±1714 | 240.614 | 2.29 | 666.502 | 6.35 |
| ALMI (kg/m^2^) | 5.5±0.9 | 0.088 | 1.60 | 0.243 | 4.44 |
| **Trunk** |  |  |  |  |  |
| BMC (g) | 549±98 | 9.721 | 1.77 | 26.927 | 4.91 |
| Lean (g) | 17300±2707 | 268.948 | 1.56 | 744.987 | 4.31 |
| Fat (g) | 11420±2657 | 284.906 | 2.50 | 789.190 | 6.91 |
| PBF (%) | 38.7±5.1 | 0.884 | 2.28 | 2.448 | 6.32 |

**Abbreviations:** ALM, appendicular lean mass; ALMI, appendicular lean mass index; BMC, bone mineral content; BMD, bone mineral density; CV, correlation of variation; LSC, least significant change; PBF, percentage body fat; RMS SD, root mean square standard deviation; SD, standard deviation.
